# Utilizing the Banana S-Adenosyl-L-Homocysteine Hydrolase Allergen to Identify Cross-Reactive IgE in Ryegrass-, Latex-, and Kiwifruit-Allergic Individuals

**DOI:** 10.3390/ijms25115800

**Published:** 2024-05-26

**Authors:** Tatjana Đurašinović, Zorana Lopandić, Isidora Protić-Rosić, Tina Ravnsborg, Gordan Blagojević, Lidija Burazer, Ole N. Jensen, Marija Gavrović-Jankulović

**Affiliations:** 1 Institute of Medical Biochemistry, Military Medical Academy, 11000 Belgrade, Serbia; tatjana.djurasinovic@gmail.com; 2 Institute for Chemistry in Medicine, Faculty of Medicine, University of Belgrade, 11000 Belgrade, Serbia; zorana.lopandic@med.bg.ac.rs; 3 Faculty of Chemistry, University of Belgrade, 11000 Belgrade, Serbia; proticrosic@chem.bg.ac.rs; 4 Department of Biochemistry and Molecular Biology, University of South Denmark, 5230 Odense, Denmark; 5 Institute of Virology, Vaccines and Sera “Torlak”, 11000 Belgrade, Serbia; gblagojevic@torlak.rs (G.B.); lburazer@torlak.rs (L.B.)

**Keywords:** S-adenosyl-L-homocysteine hydrolase (SAHH), banana, kiwifruit, latex, ryegrass, panallergen, cross-reactivity, B-cell epitopes, T-cell epitopes

## Abstract

Food allergies mediated by specific IgE (sIgE) have a significant socioeconomic impact on society. Evaluating the IgE cross-reactivity between allergens from different allergen sources can enable the better management of these potentially life-threatening adverse reactions to food proteins and enhance food safety. A novel banana fruit allergen, S-adenosyl-L-homocysteine hydrolase (SAHH), has been recently identified and its recombinant homolog was heterologously overproduced in *E. coli*. In this study, we performed a search in the NCBI (National Center for Biotechnology Information) for SAHH homologs in ryegrass, latex, and kiwifruit, all of which are commonly associated with pollen-latex-fruit syndrome. In addition, Western immunoblot analysis was utilized to identify the cross-reactive IgE to banana SAHH in the sera of patients with a latex allergy, kiwifruit allergy, and ryegrass allergy. ClustalOmega analysis showed more than 92% amino acid sequence identity among the banana SAHH homologs in ryegrass, latex, and kiwifruit. In addition to five B-cell epitopes, in silico analysis predicted eleven T-cell epitopes in banana SAHH, seventeen in kiwifruit SAHH, twelve in ryegrass SAHH, and eight in latex SAHH, which were related to the seven-allele HLA reference set (HLA-DRB1*03:01, HLA-DRB1*07:01, HLA-DRB1*15:01, HLA-DRB3*01:01, HLA-DRB3*02:02, HLA-DRB4*01:01, HLA-DRB5*01:01). Four T-cell epitopes were identical in banana and kiwifruit SAHH (positions 328, 278, 142, 341), as well as banana and ryegrass SAHH (positions 278, 142, 96, and 341). All four SAHHs shared two T-cell epitopes (positions 278 and 341). In line with the high amino acid sequence identity and B-cell epitope homology among the analyzed proteins, the cross-reactive IgE to banana SAHH was detected in three of three latex-allergic patients, five of six ryegrass-allergic patients, and two of three kiwifruit-allergic patients. Although banana SAHH has only been studied in a small group of allergic individuals, it is a novel cross-reactive food allergen that should be considered when testing for pollen-latex-fruit syndrome.

## 1. Introduction

An allergy is a chronic, non-communicable disease that affects 25% of the world’s population and significantly affects modern society on a socioeconomic level [1]. Allergens are innocuous environmental compounds found in a variety of plant and animal materials and cause type I hypersensitivity in susceptible individuals. The clinical manifestations of allergic reactions can vary depending on the route of exposure, and they range from mild itching to potentially fatal anaphylaxis [2,3]. When exposed to an allergen, non-allergic individuals produce an IgG response. However, in the allergic subject, the immune response is skewed toward the production of IgE antibodies [4]. The first exposure to an allergen induces the sensitization of the immune system, including antigen-presenting cells (APCs) and B- and T cells, which leads to the production of allergen-specific IgE (sIgE), subsequently bound to high-affinity receptors (FcεRI) on mast cells and basophils. The next exposure to the allergen causes the cross-linking of FcεRI on these effector cells and the release of physiologically active mediators (histamine, leukotrienes, and prostaglandins), which induce the clinical symptoms of an allergy.

B- and T-cells are important components of humoral and cell-mediated immunity in type I hypersensitivity. Allergens are recognized by unique receptors on B-cells (BcR) and T-cells (TcR); however, their presentation occurs in quite distinct contexts. TcR recognizes an allergen fragment (T-cell epitope) on the surfaces of APCs exposed in the context of major histocompatibility complex (MHC) molecules, whereas BcR is a membrane-bound immunoglobulin that, through its antigen-binding site (paratop), recognizes complementary surfaces (B-cell epitopes) on the allergen’s native structure.

Identifying B-cell and T-cell epitopes in allergens is important in understanding the molecular basis of allergy development, creating diagnostic assays, managing safety in regard to food allergies [5], and designing vaccines based on immunodominant B-cell and T-cell epitopes [6,7] for allergen-specific immunotherapy (AIT).

Cross-reactivity between allergens from various allergen sources is a consequence of similarities in the surface topology (three-dimensional or 3D structure) of epitopes, which are recognized by a paratope of specific IgE antibodies. It is generally accepted that when the homology of the protein sequence reaches 70%, the likelihood of a cross-reaction increases [8,9].

Cross-reactivity is a characteristic of plant-derived food allergens belonging to the same protein family, which are present in grass, weed, or tree pollen. Furthermore, it has been noted that proteins with similar 3D structures, known as panallergens, can cause cross-reactions between unrelated species of plants and animals. The most significant panallergens are profilins, lipid transport proteins (LTPs), proteins from the pathogenesis-related (PR)-10 family, tropomyosins, 2S albumins, 7S globulins, parvalbumin, polcalcin, and serum albumins [10]. In addition, the majority of insect allergens cross-react with allergens found in shellfish, mollusks, and nematodes, which are cross-reacting panallergens that are extensively distributed in a variety of animal phyla [5,11,12]. Two general rules for the definition of putative allergens were established by the World Health Organization (WHO) and the Food and Agriculture Organization (FAO) [13]. The identification of putative allergens necessitates either (i) sequence identity exceeding 35% over an 80-amino-acid sequence or (ii) a minimum of six identical consecutive amino acids shared between a newly discovered food protein and a recognized allergen [14]. The in silico approach enables similarity comparisons, and it is dependent on the reference database. Mass spectrometry (MS) is a widely used technique for the characterization of novel allergens. Analyzing the amino acid sequences of proteins after enzymatic digestion followed by MS in novel foods, such as insect and microalgae flours, can help in establishing their similarity to known allergens and forecasting the likelihood of cross-reactivity [15]. The digestion of food involves numerous human proteolytic enzymes, where certain allergenic epitopes may persist unaffected [15]. For instance, αS1-casein, a milk protein allergen, exhibits over three hundred linear epitopes, some of which remain intact even after in silico digestion with key enzymes like trypsin, chymotrypsin, and pepsin [15]. Intact epitopes identified after enzymatic processing can be used as marker peptides serving as indicators for the presence of allergenic proteins in novel foods, which would aid in the prediction and management of potential allergic reactions [14,15].

The association of allergies to plant-derived foods (banana, avocado, kiwifruit, potato, etc.) and latex is called latex-fruit syndrome, and the molecular basis for the observed cross-reactivity is related to the structurally similar epitopes on different proteins that are phylogenetically closely related. Examples are the class I chitinases present in latex (Hev b 11), banana (Mus a 2), and kiwifruit (Act d Chitinase); beta-1,3-glucanase present in latex (Hev b 2) and banana (Mus a 5); profilin present in banana (Mus a 1), kiwifruit (Act d 9), and latex (Hev b 8); and nsLTP identified in banana (Mus a 3), kiwifruit (Act d 10), and latex (Hev b 12) [3,16,17,18,19,20,21].

In response to the growing demand for protein in the human diet, researchers have recently looked into alternative protein sources, such as pulses (peas, lentils, beans), algae, yeasts, insects, and lab-grown meat [22]. However, a thorough risk assessment for the prediction of food safety must be conducted prior to the introduction of proteins from such dietary alternatives into the market [23,24].

We have recently identified S-adenosyl-L-homocysteine hydrolase (SAHH) from banana fruit as a novel food allergen and a molecular marker of cross-reactivity between different plant species involved in pollen-fruit syndrome [25]. SAHH catalyzes the reversible hydrolysis of S-adenosyl-L-homocysteine (SAH) to adenosine (ADO) and L-homocysteine (HCY). L-homocysteine is further converted to S-adenosyl-methionine (SAM), acting as a methyl group donor for the modification of nucleic acids, proteins, phospholipids, etc. As a major regulator of cellular methylation reactions, SAHH is one of the most conserved enzymes in all living organisms [26].

In silico B-cell epitope mapping and 3D modeling clarified the molecular basis of banana (*Musa acuminata*) and London plane tree (*Platanus acerifolia*) pollen’s cross-reactivity by identifying five IgE-reactive epitopes in banana SAHH. Multiple sequence alignment verified the high sequence homology (above 90% identity) of SAHHs from several plant-derived foods (banana, potato, and yellow lupine) and London plane tree pollen [25]. The BLAST search of the NCBI database for the banana SAHH homologs indicates that it can be found in three well-known sources of allergens: ryegrass (*Lolium perenne*), kiwifruit (*Actinidia chinensis*), and latex (*Havea brasiliensis*).

In this study, we investigated the hypothesis that the SAHH enzyme is present in allergen extracts from latex (*Havea brasiliensis*), kiwifruit (*Actinidia deliciosa*), and ryegrass pollen (*Lolium perenne*). In addition, a cohort of people allergic to latex, kiwifruit, and grass pollen was tested for SAHH-specific IgE using recombinant banana SAHH. Additionally, the landscape and resemblance of the in silico predicted B-cell and T-cell epitope maps in the SAHH allergens were examined.

## 2. Results

### 2.1. Protein Profiling and SAHH Activity in Allergen Extracts

**Figure 1:**
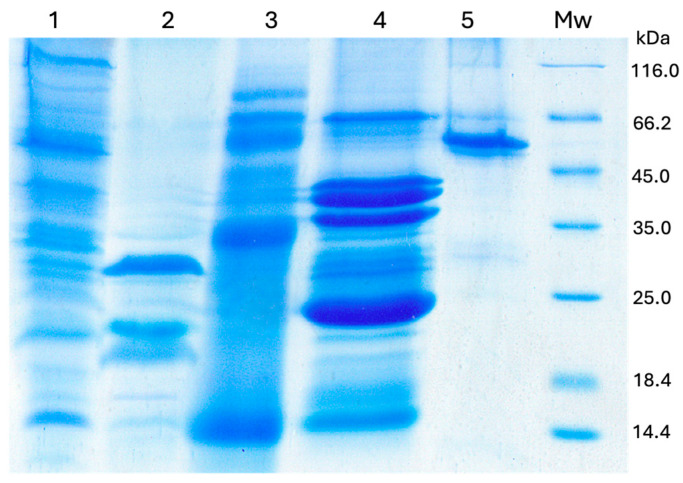
Protein profiles of four allergen extracts obtained by SDS-PAGE (14% gel): (1) banana, (2) kiwifruit, (3) ryegrass, (4) latex, (5) rSAHH and Mw (molecular weight markers).

The protein profiles of banana, kiwifruit, ryegrass, and latex allergen extracts were analyzed in SDS-PAGE (Figure 1). Banana and ryegrass revealed a broad range of proteins with molecular weights from 116 kD to 10 kD. Kiwifruit extract contains proteins in the range of 67 kD to 10 kD, with predominant bands of about 28 kD and 24 kD, while latex extract contains proteins in the range of 67 kD to 14.4 kD with the dominant bands around 40 kD, 37 kD, and 24 kD. Recombinant banana SAHH revealed a molecular mass of about 55 kD.

Ellman’s reagent was used to test for the presence of SAHH in the prepared allergen extracts. SAHH activity was indeed detected in each of the four analyzed extracts, with an adjusted protein concentration of 0.25 mg/mL. The SAHH activity was determined to be 0.85 U/mL in the banana extract, 0.25 U/mL in the kiwifruit extract, 0.46 U/mL in the ryegrass extract, and 0.219 U/mL in the latex extract.

### 2.2. Isolation of Recombinant Banana SAHH

**Figure 2:**
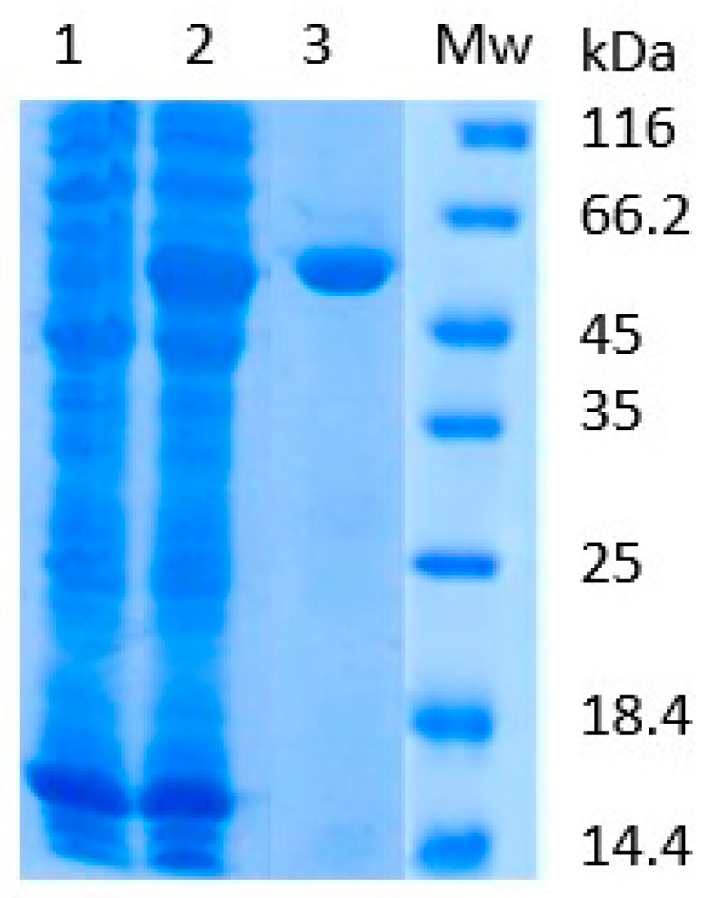
rSAHH purification from *E. coli* cell lysate was analyzed using SDS-PAGE: (1) cell lysate before addition of IPTG, (2) cell lysate after overnight expression of rSAHH, (3) rSAHH purified by IMAC, (Mw) molecular weight markers.

Recombinant SAHH was produced in *E. coli* BL21 cells as a soluble protein. Protein expression was induced using 0.5 mM isopropyl-beta-D-thiogalactoside (IPTG). After overnight expression, the protein was isolated from the cell lysate by immobilized metal affinity chromatography (IMAC). Protein purification was analyzed by SDS-PAGE (Figure 2).

### 2.3. Mass Spectrometry Analysis of Recombinant Banana SAHH

**Figure 3:**
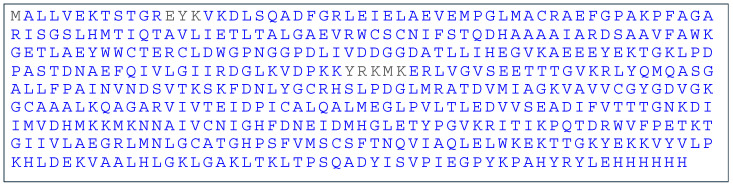
Mass spectrometry verified 98% of the recombinant banana SAHH’s amino acid sequence; peptides confirmed by MS are indicated in blue.

**Figure 4:**
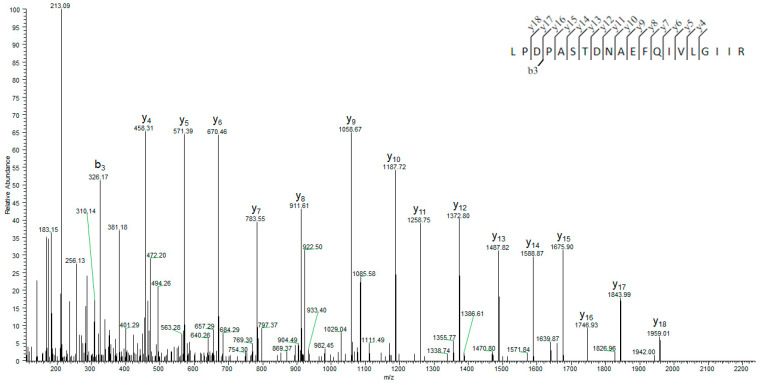
MSMS spectrum of the precursor *m*/*z* 1085.081 with y and b ions assigned by Mascot.

The analysis of the rSAHH peptides on Mascot Server™ confirmed a 98% match to the protein sequence. In Figure 3, the sequence coverage is illustrated, and the list of detected SAHH peptides is shown in Supplementary Table S1. In Figure 4, a representative tandem mass spectrum of a peptide from the banana SAHH sequence is presented.

### 2.4. Bioinformatics Analysis of SAHH Amino Acid Sequence

**Figure 5:**
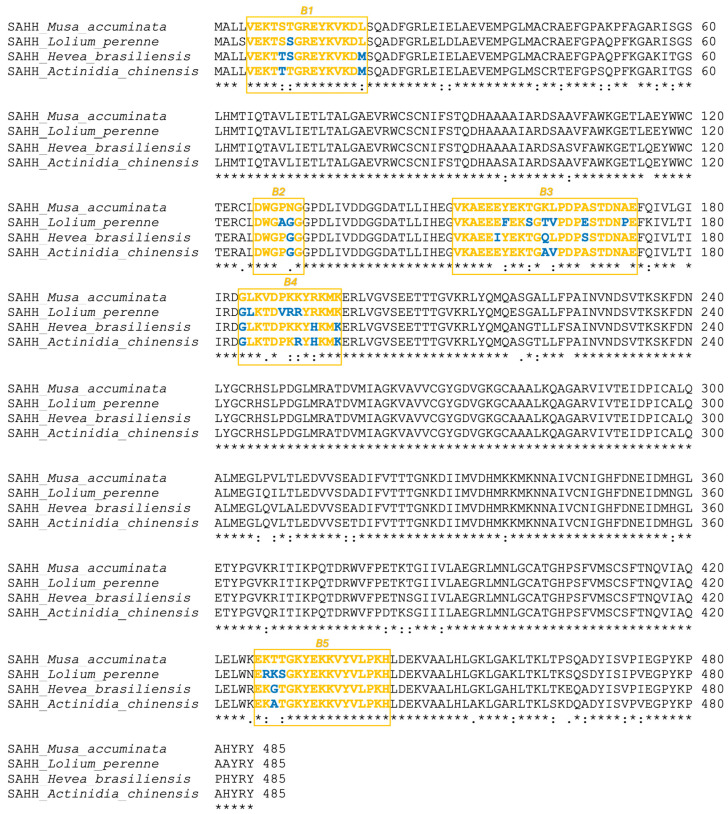
The molecular basis of SAHH IgE cross-reactivity: sequence alignment of SAHH allergens from banana, ryegrass, latex, and kiwifruit. Yellow boxes represent B-cell epitopes; conserved sequence (*), conservative mutations (:), semi-conservative mutations (.), and non-conservative mutations ( ).

Using the NCBI database’s BLAST search, the amino acid sequence homologs to banana SAHH (XP_009415939.1) from kiwifruit (PSS23942.1), ryegrass (XP_051195844.1), and latex (XP_021689860.1) were identified. All analyzed SAHH proteins were 485 amino acids in length, and their theoretical molecular weights were 53,059.30 Da for banana, 53,312.29 Da for kiwifruit, 53,256.23 Da for ryegrass, and 53,271.29 Da for latex, respectively. The amino acid sequence homology among the analyzed SAHHs was above 92% (Figure 5).

### 2.5. Profiling of B-Cell Epitopes

**Figure 6:**
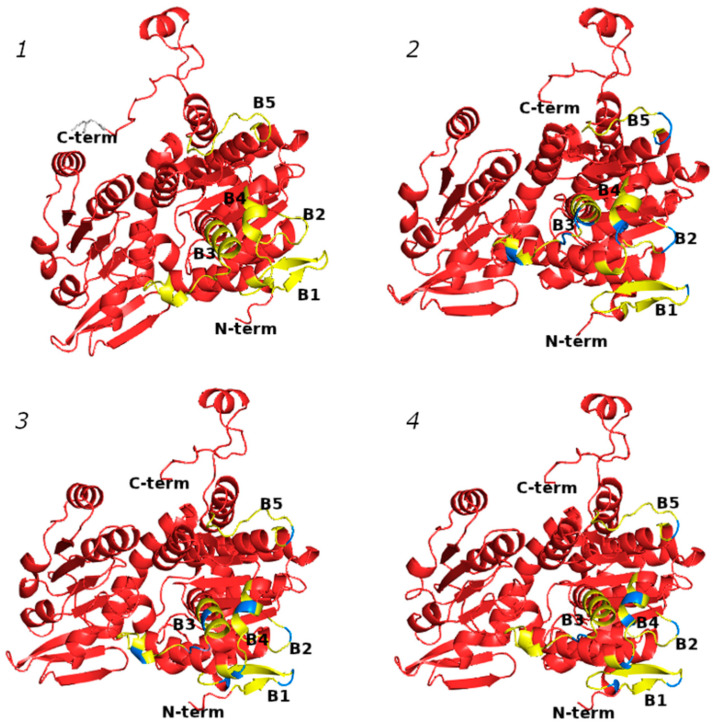
In silico prediction of B-cell epitopes in SAHH 3D structure: (1) banana, (2) ryegrass, (3) latex, and (4) kiwifruit. B-cell epitopes are marked in yellow; differences in amino acid sequence are marked in blue.

As was previously performed by using Bepitope (http://bepitope.ibs.fr/, accessed on 15 February 2022) and the BepiPred 2.0 server [25], the B-cell epitopes of SAHHs were confirmed for the panel of analyzed proteins. In silico analysis confirmed the presence of five highly conserved linear B-cell epitopes across all analyzed SAHHs: B1 (5–19), B2 (126–131), B3 (151–173), B4 (184–196), and B5 (426–437). All of the epitopes (Figure 6) were well exposed on the SAHH surface, which is a characteristic shared by immunodominant epitopes from other food allergens [7,27,28,29].

### 2.6. Profiling of T-Cell Epitopes

In the sensitization phase of type I hypersensitivity, professional antigen-presenting cells (APCs), such as dendritic cells, are specialized to uptake exogenous antigens (allergens) and to present antigen peptides to CD4^+^ T-cells in the context of MHC class II molecules (peptide–MHC class II complex). Peptide fragments, usually ranging from 13 to 17 amino acids in length, are attached to MHC class II molecules and are exposed on the cell surface. With the NetMHCIIpan 4.0 program, the T-cell epitopes derived from banana SAHH were in silico mapped. Using the reference set of MHC II molecules and a 1% threshold for strongly binding peptides, eleven T-cell epitopes with a peptide length of fifteen amino acids were found. The list of peptides is given in Table 1. The same analysis showed the presence of seventeen T-cell epitopes in kiwifruit SAHH, twelve T-cell epitopes in ryegrass SAHH, and eight T-cell epitopes in latex SAHH. The list of T-cell epitopes is given in Supplementary Table S2. Four T-cell epitopes are identical in banana and kiwifruit SAHH (positions 328, 278, 142, and 341) and banana and ryegrass SAHH (positions 278, 142, 96, and 341). Two T-cell epitopes are identical in banana and latex SAHH (positions 278 and 341). Differences in the amino acid sequences of the cross-reactive T-cell epitopes compared to those found in banana SAHH are highlighted in red in Table 2, Table 3 and Table 4.

### 2.7. Detection of SAHH Cross-Reactive IgE in Ryegrass-, Latex-, and Kiwifruit-Allergic Individuals

**Figure 7:**
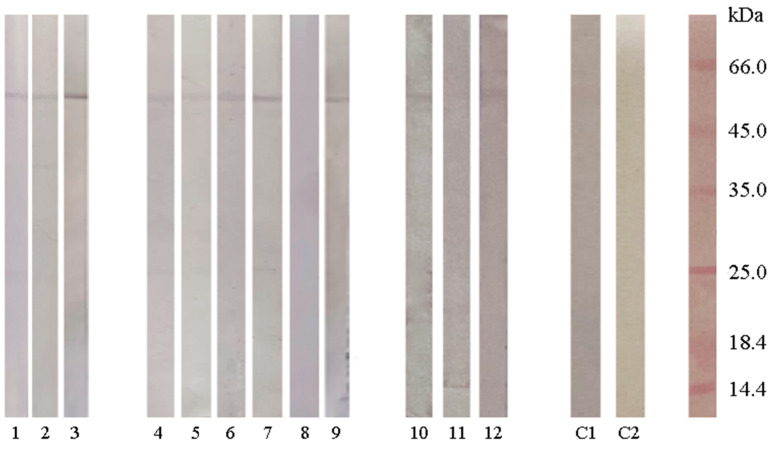
Cross-reactive IgE was detected with banana rSAHH in sera from persons allergic to latex (No. 1–3), ryegrass (No. 4–9), and kiwifruit (No. 10–12); (C1) sera from person allergic to house dust mites; (C2) control of secondary antibody.

The foundation for the evaluation of whether sera from people allergic to latex, ryegrass, and kiwifruit contained SAHH-specific IgE was the high amino acid sequence identity and B-cell epitope homology. IgE reactivity to banana SAHH was detected in three of three latex-allergic persons, five of six ryegrass-allergic persons and two of three kiwifruit-allergic persons (Figure 7).

## 3. Discussion

Data from the European Academy of Allergology and Clinical Immunology (EAACI) indicate that 60% of food allergy cases in adults coincide with inhalation allergies [10,30]. This correlation is associated with co-sensitization and cross-reactivity. Cross-reactivity manifests when an IgE-class antibody, initially generated in response to one allergen, reacts with a similar allergen even if the individual has not been exposed to the second allergen [10]. Panallergens are proteins involved in essential biological processes and are widely distributed with conserved sequences and structures [31]. These proteins contribute to numerous cross-reactions, even across phylogenetically distant and unrelated organisms [31]. Identifying panallergens is important for the improvement of component-resolved diagnostics (CRD). Incorporating these cross-reactive allergens in CRD enables physicians to recommend avoiding specific foods or airborne substances that exhibit cross-reactivity [32]. The issue of cross-reactivity becomes notably significant when novel allergens are taken into account. In response to the increasing demand for protein in the human diet, there has been consideration of alternative protein sources such as plants, as well as more unconventional options like algae, insects, and lab-grown meat [22]. LC-MS/MS coupled with the in silico analysis of the proteomes of algae and insects revealed that they predominantly consist of panallergens with significant sequence homology with already identified allergens [5,22,23]. This indicates the importance of identifying new panallergens to create a database that would facilitate the recognition of allergens from new food sources. Our group previously identified SAHH from banana fruit as a novel plant panallergen [25]. The present investigation verified that the tested allergen extracts, namely banana, kiwifruit, ryegrass, and latex, all demonstrated SAHH enzymatic activity. The banana extract demonstrated the highest SAHH activity (0.85 U/mL), followed by ryegrass (0.46 U/mL), and comparable levels were observed in kiwifruit and latex (0.25 U/mL and 0.219 U/mL).

The literature suggests that identity in the amino acid sequences between cross-reactive allergens is higher than 70% [33]. However, it is important to emphasize the importance of shared B-cell and T-cell epitope sequences [34,35]. While the potential for cross-reactivity among food allergens due to shared linear or conformational epitopes raises concerns, recent findings suggest that conformational epitopes may not exert a significant influence, as reported by Curin et al. [36]. The sequence homology between the examined SAHHs was found to be higher than 92%. In addition, five highly conserved linear B-cell epitopes were confirmed by in silico analysis. The results indicated that SAHHs mediated the IgE cross-reactivity among the tested allergen sources. In silico analysis revealed the presence of cross-reactive linear B-cell epitopes among other food allergen groups, including shellfish, house dust mites, and cockroaches, which exhibited a correlation with IgE immunoreactivity, as confirmed by dot-blot assays [37]. By identifying common T-cell epitopes among panallergens, CRD can provide insights into cross-reactivity patterns and improve the precision of allergy diagnosis [38]. In addition, a non-IgE response can be triggered by T-cells, emphasizing the importance of identifying pairs of peptides that can bind to class II MHC molecules [39]. The predominant cause and mechanism of T-cell cross-reactivity frequently arise from the sequence similarity of the T-cell cross-reactive peptide residues found among closely related allergen sources [40]. The presence of cross-reactive epitopes in allergen preparations utilized for allergen immunotherapy is crucial in ensuring the safety and effectiveness of the treatment, as well as for its goal of achieving desensitization and clinical tolerance [41]. A recent study revealed that individuals undergoing grass sublingual immunotherapy reported favorable tolerance toward plant-based food [23]. Control over cross-reactive epitopes in food allergy immunotherapy can aid in desensitization to multiple food sources, leveraging well-characterized T-cell epitopes [41]. In addition, cross-reactive epitopes can help to reduce the number of allergens without compromising the efficacy of the therapy [33,42]. Current advances in immunoinformatic tools offer potential solutions by aiding in the identification of specific shared cross-reactive peptide sequences covering a range of different allergen sources, thereby addressing the challenge of designing a single peptide- or protein-based candidate [41]. Using an in silico approach, we have identified T-cell epitopes with a reference set of MHC class II molecules in SAHHs from banana, kiwifruit, ryegrass, and latex. Compared to the SAHH from banana, the SAHHs from kiwifruit and ryegrass contained four identical T-cell epitopes at positions 328, 278, 142, and 341 and 278, 142, 96, and 341, respectively, while the SAHH from latex revealed two identical T-cell epitopes at positions 278 and 341. These findings suggest that peptides 278 and 341, used either alone or in combination with peptides 328, 96, and 142, could represent potential candidates for allergen immunotherapy (AIT), capable of addressing allergies to all four cross-reactive sources. Utilizing both bioinformatic and serologic techniques is an important approach for the confirmation of the IgE-reactive epitopes of allergens [43]. Immunoblot analysis revealed the specific reactivity of SAHH from banana with sera obtained from patients allergic to kiwifruit, ryegrass, and latex. This observation suggests potential cross-reactivity between these allergens and highlights the importance of investigating shared epitopes and allergenic components among different sources. Such cross-reactivity underscores the need for comprehensive diagnostic approaches and therapeutic strategies in managing allergies associated with these diverse allergens. Overall, the observation of SAHH reactivity across multiple allergens highlights the complexity of allergic responses and the need for comprehensive diagnostic and therapeutic approaches that consider both IgE-mediated and T-cell-mediated mechanisms of cross-reactivity.

## 4. Materials and Methods

### 4.1. Preparation of Allergen Extracts

Banana and kiwifruit allergen extracts were prepared according to the previously published procedure [44]. Ryegrass pollen (*Lolium perenne*) extract was prepared following a previously published procedure [44,45]. In brief, one gram of ryegrass pollen (Institute of Virology, Vaccines and Sera, “Torlak”, Belgrade, Serbia) was extracted with 10 mL of phosphate-buffered saline (PBS) overnight at 4 °C with constant stirring. The suspension was centrifuged (5 min, 10,000× *g*), and the supernatant was collected and stored at −20 °C until use. The protein concentration in the kiwifruit (0.886 mg/mL), banana (0.539 mg/mL), and ryegrass (1.031 mg/mL) allergen extracts was determined using the bicinchonic acid (BCA) assay. The latex protein extract (0.244 mg/mL) was provided by the Institute of Virology, Vaccines and Sera, “Torlak”, Belgrade, Serbia. The protein profiles of the four allergen extracts were analyzed by SDS-PAGE [46]. Electrophoretic analysis was repeated three times.

### 4.2. Detection of SAHH Activity in Allergen Extracts

The SAHH activity was determined in four allergen extracts (protein concentrations adjusted to 0.25 mg/mL) following the procedure of [47]. The SAHH activity was calculated by using the molar absorption coefficient of reduced Ellman’s reagent of 13,600 L/mol cm under assay conditions. One unit of SAHH hydrolytic activity (IU) was defined as the amount of enzyme that produced 1 µmol of L-homocysteine per minute under assay conditions. The experiments were performed in three biological replicates in triplicate.

### 4.3. Recombinant SAHH Protein Expression and Purification

The recombinant SAHH (rSAHH) from banana fruit was produced according to the previously described procedure. In brief, the cloning strategy was to insert the SAHH gene (NCBI XM_009417664) into the pET23b vector with the Nde*I* and Xho*I* (Thermo Fisher Scientific, Waltham, MA, USA) restriction enzymes with the 6His tag on the C-terminus [25]. The rSAHH was heterologously overproduced in *Escherichia coli* BL21(DE3) cells, by adding 0.5 mM IPTG (Thermo Fisher Scientific, Waltham, MA, USA). The protein was purified by immobilized metal affinity chromatography (IMAC, TALON Superflow Affinity Resin, 1 mL; GE Healthcare, Uppsala, Sweden). The protocol for rSAHH purification was carried out four times. Protein homogeneity was verified by SDS-PAGE and biological activity by an SAHH assay (61.97 IU/mg).

### 4.4. Mass Spectrometry Analysis of Recombinant SAHH

The digests of purified rSAHH were subjected to mass spectrometric analysis using the Mascot Server™ database version 2.8.2. Coomassie-stained protein bands were excised and prepared for trypsin digestion following the previously published procedure of Shevchenko et al. [48]. Protein bands were destained in 100 mM ammonium bicarbonate/acetonitrile (1:1, vol/vol) solution and reduced with 10 mM dithiothreitol (DTT) solution (30 min at 56 °C). Subsequently, the protein bands were alkylated with 55 mM iodacetamine (Sigma-Aldrich, St. Louis, MO, USA) solution and digested with trypsin (Promega V5113, Madison, WI, USA) overnight at 37 °C [48]. The samples were analyzed on an EASY-nLC coupled in line to an Orbitrap Q-Exactive HF Mass Spectrometer (Thermo Fisher Scientific, San Jose, CA, USA). The samples were loaded in solvent A (0.1% formic acid) onto a 2-column setup consisting of a 100 ID, a 3 cm precolumn packed with Reprosil-Pur 120 C18-AQ (5 µm; Dr. Maisch), and a 75 ID, 18 cm analytical column packed with Reprosil-Pur 120 C18-AQ (3 µm; Dr. Maisch). Peptides were eluted with a gradient of solvent B (95% acetonitrile, 0.1% formic acid), 2% B to 34% B in 10 min, 34% B to 100% B in 5 min, and 100% B for 8 min. MS data were recorded with a resolution of 60,000 and a scan range of 350–1600 *m*/*z*. MS/MS was obtained as the top 12 with a resolution of 30,000, an isolation window of 1.2 *m*/*z*, and 3 s dynamic exclusion. The resulting experimental MS/MS spectra were analyzed using the Mascot Server^TM^. The search criteria included a 5 ppm tolerance for precursor ions and a 0.1 Da tolerance for fragment ions. The type of search conducted was an MS/MS ion search, with carbamidomethyl (cysteine) specified as a fixed modification and oxidation (methionine) as a variable modification.

### 4.5. Bioinformatics Analysis, 3D Modeling, and In Silico B- and T-Cell Epitope Mapping

An NCBI BLAST (http://www.ncbi.nlm.nih.gov/BLAST/, accessed on 15 January 2023) search was employed to identify banana SAHH (XP_009415939.1) homologs from kiwifruit (PSS23942.1), ryegrass (XP_051195844.1), and latex (XP_021689860.1), all with E-values of 0.0 [49]. The homology among the amino acid sequences of the SAHHs was analyzed in the ClustalOmega online program (https://www.ebi.ac.uk/jdispatcher/msa/clustalo, accessed on 15 January 2023) [50]. Three-dimensional models of the SAHHs from banana, kiwifruit, ryegrass, and latex were predicted in ColabFold v1.5.2: AlphaFold 2 [51]. Using the ProtParam tool, the theoretical molecular weight of each SAHH homolog was determined (https://web.expasy.org/protparam/, accessed on 15 January 2023). The B-cell epitopes were predicted as previously described [25], employing two different tools, Bepitope (http://bepitope.ibs.fr/, accessed on 15 November 2022) and the BepiPred 2.0 server, with threshold values set at 1.5 for BepiPred and 0.5 for the Bepitope server (https://services.healthtech.dtu.dk/services/BepiPred-2.0/, accessed on 15 November 2022) [7,27,29,52,53]. The T-cell epitopes were predicted by using NetMHCIIpan 4.0 (https://services.healthtech.dtu.dk/services/NetMHCIIpan-4.0/, accessed on 21 January 2024), a freely available tool [34], for the 7-allele HLA reference set of HLA-DRB1*03:01, HLA-DRB1*07:01, HLA-DRB1*15:01, HLA-DRB3*01:01, HLA-DRB3*02:02, HLA-DRB4*01:01, HLA-DRB5*01:01, with a peptide length of 15 amino acids and a threshold for strongly binding peptides (%Rank) of 1% [54]. It uses artificial neural networks (ANNs) trained on an extensive dataset of over 500,000 measurements of binding affinity (BA) and eluted ligand mass spectrometry (EL), covering the three human MHC class II isotypes HLA-DR, HLA-DQ, and HLA-DP. The output of the model is a prediction score for the likelihood of a peptide to be naturally presented by an MHC II receptor of choice. The output also include the %rank score, which normalizes the prediction score by comparing it to the prediction of a set of random peptides.

### 4.6. Patients’ Sera

Twelve atopic patients were referred to the Clinical Allergy Unit at the Institute of Virology, Vaccines and Sera, “Torlak”, due to their clinical history of type I hypersensitivity. Written informed consent was obtained, and the study was approved by the institutional ethics committee in accordance with the Declaration of Helsinki (approval number: 40-13-1). Serum-specific IgE to banana, kiwifruit, ryegrass, and/or latex was assayed using an automated immunofluorometric method (ImmunoCAP 100; Phadia AB, Uppasla, Sweden). Results were expressed as CAP scores in classes 0–6, according to the manufacturers’ instructions (Table 5). As a control, the serum of a patient who had previously experienced a house dust mite allergy was utilized.

## 5. Conclusions

By utilizing bioinformatics tools, a landscape of B- and T-cell epitopes was obtained for the SAHHs present in banana, kiwifruit, ryegrass, and latex. Notably, the identification of highly conserved amino acid sequences, with homology exceeding 92% among the SAHH homologs, establishes SAHH as a novel molecular marker for cross-reactivity within pollen-latex-fruit syndrome. Furthermore, highly conserved T-cell epitopes may be suitable candidates for the design and development of novel allergen immunotherapies (AIT) capable of addressing allergies originating from all four cross-reactive sources. This research not only enhances our understanding of allergic cross-reactivity but also offers potential therapeutic implications for improved allergy management and treatment.

## Figures and Tables

**Table 1:** In silico predicted T-cell epitopes in banana SAHH.

Position	MHC II	Epitope	%Rank
182	DRB1_0301	RDGLKVDPKKYRKMK	0.04
96	DRB1_0301	AAAIARDSAAVFAWK	0.31
328	DRB1_0301	KDIIMVDHMKKMKNN	0.98
317	DRB1_0701	EADIFVTTTGNKDII	0.44
278	DRB1_0701	AAALKQAGARVIVTE	0.79
103	DRB1_1501	SAAVFAWKGETLAEY	0.39
142	DRB1_1501	DATLLIHEGVKAEEE	0.91
96	DRB3_0101	AAAIARDSAAVFAWK	0.09
341	DRB3_0202	NNAIVCNIGHFDNEI	0.39
170	DRB5_0101	DNAEFQIVLGIIRDG	0.57
189	DRB5_0101	PKKYRKMKERLVGVS	0.79

**Table 2:** In silico predicted cross-reactive T-cell epitopes in kiwifruit SAHH: differences in amino acid sequence to cross-reactive T-cell epitopes from banana SAHH are marked in red.

Position	MHC II	Epitope	%Rank
182	DRB1_0301	RDGLKTDPKRYHKMK	0.21
96	DRB1_0301	ASAIARDSAAVFAWK	0.31
328	DRB1_0301	KDIIMVDHMKKMKNN	0.98
317	DRB1_0701	ETDIFVTTTGNKDII	0.42
278	DRB1_0701	AAALKQAGARVIVTE	0.79
103	DRB1_1501	SAAVFAWKGETLQEY	0.38
142	DRB1_1501	DATLLIHEGVKAEEE	0.91
96	DRB3_0101	ASAIARDSAAVFAWK	0.09
341	DRB3_0202	NNAIVCNIGHFDNEI	0.39
170	DRB5_0101	DNAEFQIVLTIIRDG	0.52

**Table 3:** In silico predicted cross-reactive T-cell epitopes in ryegrass SAHH: differences in amino acid sequence to cross-reactive T-cell epitopes from banana SAHH are marked in red.

Position	MHC II	Epitope	%Rank
182	DRB1_0301	RDGLKTDVRRYRKMK	0.16
96	DRB1_0301	ASAIARDSAAVFAWK	0.31
317	DRB1_0701	DADIFVTTTGNKDII	0.44
278	DRB1_0701	AAALKQAGARVIVTE	0.79
103	DRB1_1501	SAAVFAWKGETLEEY	0.38
142	DRB1_1501	DATLLIHEGVKAEEE	0.91
96	DRB3_0101	AAAIARDSAAVFAWK	0.09
341	DRB3_0202	NNAIVCNIGHFDNEI	0.39
170	DRB5_0101	DNPEFKIVLTIIRDG	0.07

**Table 4:** In silico predicted cross-reactive T-cell epitopes in latex SAHH: differences in amino acid sequence to cross-reactive T-cell epitopes from banana SAHH are marked in red.

Position	MHC II	Epitope	%Rank
182	DRB1_0301	RDGLKTDPKKYHKMK	0.16
96	DRB1_0301	AAAIARDSASVFAWK	0.52
317	DRB1_0701	ETDIFVTTTGNKDII	0.44
278	DRB1_0701	AAALKQAGARVIVTE	0.79
103	DRB1_1501	SASVFAWKGETLQEY	0.39
96	DRB3_0101	AAAIARDSASVFAWK	0.11
341	DRB3_0202	NNAIVCNIGHFDNEI	0.39
170	DRB5_0101	DNAEFQIVLTIIRDG	0.52

**Table 5:** Allergic patients’ characteristics.

#	Sex	Years	sIgE
1.	Male	25	Latex 2.46 kU/L
2.	Male	25	Latex 48.2 kU/L
3.	Female	4	Latex 3.149 kU/L
4.	Male	11	Ryegrass 41.05 kU/L
5.	Female	28	Ryegrass 37.4 kU/L
6.	Male	19	Ryegrass 10.4 kU/L
7.	Male	13	Ryegrass 112.2 kU/L
8.	Female	29	Ryegrass 24.4 kU/L
9.	Male	13	Ryegrass 6.01 kU/L
10.	Female	39	Kiwifruit 4.31 kU/L
11.	Male	10	Kiwifruit 1.14 kU/L
12.	Male		Kiwifruit 0.67 kU/mL
c1	Male	30	House Dust Mites 4.26 kU/L
c2	Control of secondary antibodies	/	/

## Data Availability

The data presented in this study are available on request from the corresponding author.

## Supplementary Materials

The following supporting information can be downloaded at: https://www.mdpi.com/article/10.3390/ijms25115800/s1.
