# Supplementary material for: Utilizing the Banana S-Adenosyl-L-Homocysteine Hydrolase Allergen to Identify Cross-Reactive IgE in Ryegrass-, Latex-, and Kiwifruit-Allergic Individuals
Source: Int J Mol Sci. 2024 May 26;25(11):5800. doi: 10.3390/ijms25115800 (PMC11171677; doi:10.3390/ijms25115800)
Supplement: Supplementary file 1 [file ijms-25-05800-s001.zip › Supplementary Table S2.pdf]

Supplementary Table S2:

A) List of T-cell epitopes in kiwifruit SAHH.

Position	MHC II	Epitope	%Rank
182	DRB1_0301	RDGLKTDPKRYHKMK	0.21
375	DRB1_0301	TDRWVFPDTKSGIII	0.28
96	DRB1_0301	ASAIARDSAAVFAWK	0.31
328	DRB1_0301	KDIIMVDHMKKMKNN	0.98
154	DRB1_0701	EEEYEKTGAVPDPAS	0.13
317	DRB1_0701	ETDIFVTTTGNKDII	0.42
482	DRB1_0701	HYRYADYISVPIEGP	0.68
278	DRB1_0701	AAALKQAGARVIVTE	0.79
103	DRB1_1501	SAAVFAWKGETLQEY	0.38
482	DRB1_1501	HYRYADYISVPIEGP	0.73
142	DRB1_1501	DATLLIHEGVKAEEE	0.91
96	DRB3_0101	ASAIARDSAAVFAWK	0.09
459	DRB3_0101	LTKLSKDQADYISVP	0.74
341	DRB3_0202	NNAIVCNIGHFDNEI	0.39
455	DRB4_0101	LGARLTKLSKDQADY	0.95
170	DRB5_0101	DNAEFQIVLTIIRDG	0.52
420	DRB5_0101	QLELWKEKATGKYEK	0.70

B) List of T cell epitopes in latex SAHH.

Position	MHC II	Epitope	%Rank
182	DRB1_0301	RDGLKTDPKKYHKMK	0.16
96	DRB1_0301	AAAIARDSASVFAWK	0.52
317	DRB1_0701	EADIFVTTTGNKDII	0.44
278	DRB1_0701	AAALKQAGARVIVTE	0.79
103	DRB1_1501	SASVFAWKGETLQEY	0.39
96	DRB3_0101	AAAIARDSASVFAWK	0.11
341	DRB3_0202	NNAIVCNIGHFDNEI	0.39
170	DRB5_0101	DNAEFQIVLTIIRDG	0.52

C) List of T-cell epitopes in ryegrass SAHH

Position	MHC II	Epitope	%Rank
182	DRB1_0301	RDGLKTDVRRYRKMK	0.16
96	DRB1_0301	AAAIARDSAAVFAWK	0.31
317	DRB1_0701	DADIFVTTTGNKDII	0.44
278	DRB1_0701	AAALKQAGARVIVTE	0.79
154	DRB1_0701	EEEFEKSGTVPDPES	0.84
103	DRB1_1501	SAAVFAWKGETLEEY	0.38

356	DRB1_1501	DMNGLETYPGVKRIT	0.90
142	DRB1_1501	DATLLIHEGVKAEEE	0.91
96	DRB3_0101	AAAIARDSAAVFAWK	0.09
341	DRB3_0202	NNAIVCNIGHFDNEI	0.39
21	DRB4_0101	QADFGRLELDLAEVE	0.58
170	DRB5_0101	DNPEFKIVLTIIRDG	0.07
